# Urany-Less Low Voltage Transmission Electron Microscopy: A Powerful Tool for Ultrastructural Studying of Cyanobacterial Cells

**DOI:** 10.3390/microorganisms11040888

**Published:** 2023-03-29

**Authors:** Katerina Mrazova, Jaromir Bacovsky, Zuzana Sedrlova, Eva Slaninova, Stanislav Obruca, Ines Fritz, Vladislav Krzyzanek

**Affiliations:** 1 Institute of Scientific Instruments of the Czech Academy of Sciences, v.v.i., Kralovopolska 147, 612 64 Brno, Czech Republic; mrazova@isibrno.cz; 2 Delong Instruments a.s., Palackeho Trida 3019/153 b, 612 00 Brno, Czech Republic; jaromir.bacovsky@delong.cz; 3 Department of Food Chemistry and Biotechnology, Faculty of Chemistry, Brno University of Technology, Purkynova 118, 612 00 Brno, Czech Republic; zuzana.sedrlova@vut.cz (Z.S.); xcslaninovae@fch.vut.cz (E.S.); obruca@fch.vut.cz (S.O.); 4 Institute of Environmental Biotechnology, Department of Agrobiotechnology, IFA-Tulln, University of Natural Resources and Life Sciences, Konrad-Lorenz-Strase 20, 3430 Tulln an der Donau, Austria; ines.fritz@boku.ac.at

**Keywords:** low voltage electron microscopy, uranyl acetate, contrasting agents, transmission electron microscopy, *Synechocystis*, polyhydroxyalkanoates

## Abstract

Sample preparation protocols for conventional high voltage transmission electron microscopy (TEM) heavily rely on the usage of staining agents containing various heavy metals, most commonly uranyl acetate and lead citrate. However high toxicity, rising legal regulations, and problematic waste disposal of uranyl acetate have increased calls for the reduction or even complete replacement of this staining agent. One of the strategies for uranyless imaging is the employment of low-voltage transmission electron microscopy. To investigate the influence of different imaging and staining strategies on the final image of cyanobacterial cells, samples stained by uranyl acetate with lead citrate, as well as unstained samples, were observed using TEM and accelerating voltages of 200 kV or 25 kV. Moreover, to examine the possibilities of reducing chromatic aberration, which often causes issues when imaging using electrons of lower energies, samples were also imaged using a scanning transmission electron microscopy at 15 kV accelerating voltages. The results of this study demonstrate that low-voltage electron microscopy offers great potential for uranyless electron microscopy.

## 1. Introduction

To date, transmission electron microscopy (TEM) is commonly the number one method used for observing both the shape and intracellular space of cells [1,2]. The current microscopes have revealed specimen details right down to the atomic structure [3,4], yet the resolution is not the only parameter used to evaluate image quality. The second parameter, which is of equal importance as the spatial resolution, is image contrast. This parameter starts to be highly crucial, especially when imaging biological sections or other specimens composed of light elements. 

Briefly, the incident electrons in the transmission electron microscope that interact with the sample are dependent on the thickness and chemical composition of the matter [5,6]. Since biological samples are composed of light elements and are also embedded in carbon-based resins, the interactions between the primary electron beam and the sample are not sufficient to provide satisfactory contrasts [6]. 

There are several ways to deal with low image contrast. Conventional imaging of biological samples requires specimen staining. [7,8]. However, such sample preparation for TEM includes the use of various toxic substances, namely osmium tetroxide, or organic salts of heavy metals, such as uranium and lead, which are used as postfixation or contrasting agents [7,9]. Specifically high toxicity, together with rising legal regulations and problematic waste disposal of uranyl acetate, is regularly used as a contrasting agent for ultrathin sections alongside a negative staining agent, have raised the need for changes in conventional biological sample preparation procedures for TEM [10,11]. 

Several strategies on how to replace uranyl acetate are being tested. Salts of the lanthanoid series of elements pose as the most frequently suitable substitutes [11,12,13]. Interestingly, some publications also suggest oolong tea extract as a staining agent for ultrathin sections [14,15,16]. A different approach, how to increase the contrast of the image without heavy metal staining, is to alter the imaging technique itself. It can be realized by construction improvements of electron optics, such as a microscope that can be equipped with a phase plate [17,18], or another strategy is to lower the accelerating voltage of the primary electron beam [19,20]. 

To explain the origin of the image contrast, the previously mentioned electron interactions with the sample have to be considered. There are several mechanisms involved in the contrast formation of TEM images, connected to the various scenarios of the incident electron beam interaction with the sample. Electrons can be elastically or inelastically scattered or they can go through the sample without any interaction, while all of these phenomena manifest differently in the final image. Electrons transmitted through the sample without any interaction contribute to the bright background of the image. Elastically scattered electrons are filtered by the apertures and, thus, increase the image contrast. The most problematic are inelastically scattered electrons, which lose some energy interacting with the sample, because they broaden the energy spectrum and, thus, bring an additional contribution to the chromatic aberration. The probability of each kind of interaction is dependent on the value of the cross-section, which is specific to a particular type of interaction, sample composition, and electron energy [5,6].

For conventional transmission electron microscopes that use accelerating voltages between 60 and 300 kV [6,19], phase contrast is essential [6]. However, going to the lower beam energy, phase contrast is no longer as necessary and the scattering contrast becomes the most important component of the contrast. Reducing the electron beam energy leads to higher scattering and, thus, to higher image contrasts. In other words, the intensity of the electron interaction with the sample is dependent on the thickness and chemical composition of the matter [6,20,21]. 

**Figure 1:**
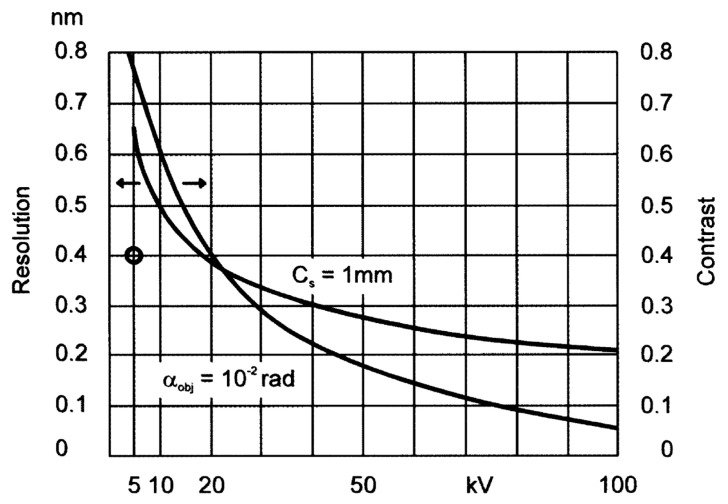
The dependence of contrast and resolution on the accelerating voltage of the primary electron beam for TEM imaging of 20 nm carbon layers [20,27].

The dependence of the contrast of the resulting image on the accelerating voltage of the primary electron beam in Figure 1 shows that the contrast of the image decreases with higher electron energies, while the resolution increases [20]. As was described for various layers of polymers and polymer blends, imaging using lower energies provides the possibility to even distinguish areas of different compositions in carbon-based samples [20,22,23].

Studies employing low-voltage transmission electron microscopy for the examination of biological samples have already proven the benefits of imaging at an accelerating voltage of 5 kV [19,24]. However, using such low energies comes together with the need for even thinner samples than the conventional ~70 nm. Specifically, to observe the pancreatic tissue of a rat, Bendayan et al. chemically fixed the samples using glutaraldehyde without any postfixation procedure, while for comparison osmificated samples were also prepared. Samples embedded in Epon resin were cut to ultrathin sections of 30–40 nm thickness. Observation using a low voltage electron microscope (LVEM) revealed that imaging using a 5 kV electron beam provided sufficient contrast of the resulting image and was able to expose structures, which were otherwise covered by osmium or other heavy metals. [24] Nevertheless, the preparation of sections 30–40 nm thin is quite challenging for common users of ultramicrotome, even when using a diamond knife [25]. However, using moderately higher accelerating voltages of 25 kV, the electrons of the primary electron beam had sufficient energy to penetrate the thicker common sections of 70 nm [26].

The model organism of choice, with well-described intracellular structures for our study, was the unicellular cyanobacterium of PHA-producing strain *Synechocystis* sp., PCC 6803. Cyanobacteria belong to organisms significant in both the fields of science and industry. They are capable of growing in diverse environments and can adapt to various adverse growth conditions (e.g., hypersaline environment [28], high or low temperatures [29,30], UV irradiation [31], etc.). Moreover, many cyanobacterial strains pose as producers of biotechnologically valuable substances [32,33,34]. Specifically, members of the genus *Synechocystis* are, along with some heterotrophic microorganisms, capable of producing polyhydroxyalkanoates (PHAs), which are polyesters of hydroxy acids that form granules inside various microbial cells [35,36,37]. In addition to serving as storage for carbon and energy, PHAs have a significant role in the enhanced robustness of cells and, therefore, in the capability of the cells to survive harsh environmental conditions [38,39]. 

The ability to cope with unfavorable environmental conditions often comes together with the possibility of morphological changes to the cells, as was previously proven for both heterotrophic bacteria and cyanobacteria. For various microorganisms, the observed changes in the shape and size of cells, as well as the formation of precipitates and other changes in the cytoplasm were related to exposure to various stress conditions [40,41,42]. 

In our study, we aimed to prove that an electron beam of 15–25 kV was still capable of providing high contrast for lighter elements and, therefore, it was possible to observe biological samples without the necessity of heavy metals staining. Samples were fixed using the high-pressure freezing method, followed by freeze substitution. Then, samples were observed using TEMs operating at 200 kV and 25 kV accelerating voltages. Both microscopes were used for the analysis of the samples stained using salts of uranyl acetate and lead citrate alongside samples without staining with heavy metals. To demonstrate the possibility of how to minimize chromatic aberration at lower accelerating voltages, imaging with a scanning transmission electron microscope (STEM) mode was also performed.

## 2. Materials and Methods

### 2.1. Microorganisms and Their Cultivation

In this study cultures of the cyanobacterium *Synechocystis* sp., PCC 6803, were obtained from the Pasteur culture collection (Paris, France) and used. Cultures were cultivated in a mineral medium based on BG-11 [43,44]. The content of nitrogen and phosphorus was adjusted to enable early growth of biomass followed by starvation of nitrogen and phosphorus to induce PHA production. Cultures in Erlenmeyer flasks were cultivated in a transparent box with controlled air flow, temperature, and daylight illumination simulation using a 16/8 h day/night cycle of illumination. 

### 2.2. Sample Preparation for Electron Microscopy

Cyanobacterial cultures were centrifuged for 4 min at 4000 rpm and processed using cryogenic methods for sample preparation. The cell pellet was pipetted on 3 mm Au/Cu carriers type A with 1% solution of soy lecithin in chloroform and covered with the flat side of 3 mm Au/Cu carrier type B. Samples were fixed using high-pressure freezing (EM ICE, Leica Microsystems, Vienna, Austria), followed by freeze substitution (EM AFS2, Leica Microsystems, Vienna, Austria). The substitution solution contained 1.5% OsO_4_ in acetone and the procedure was set to −90 °C for 72 h, then, the samples were warmed up to −20 °C for 24 h and the procedure finished, with the final phase at 4 °C for 18 h, as previously described [45]. Fixed samples were infiltrated with epoxy resin (Epoxy Embedding Medium kit, Sigma Aldrich, Darmstadt, Germany ) and cured for 48 h at 62 °C. Embedded samples were cut into ultrathin sections (~75 nm) using a diamond knife (Ultra 45°, DiATOME, Nidau, Switzerland) and ultramicrotome (EM UC7, Leica Microsystems, Vienna, Austria). Half of the sections were observed without any poststaining procedures, while the other half were stained using conventional staining agents: uranyl acetate and lead citrate. 

### 2.3. Electron Microscopy

Cyanobacterial cells were observed using various types of electron microscopes. As previously mentioned, the conventional transmission electron microscope operates within the range of 60–300 kV accelerating voltages for the electron beam [6,19]. In our study, we compared two different transmission electron microscopes using different accelerating voltages for the electron beam: Talos F200C operating at 200 kV and LVEM 25 operating at 25 kV. Moreover, conventional TEM imaging requires staining of ultrathin sections commonly using uranyl acetate and lead citrate [7,8], which should not be necessary for low-voltage imaging [24]. Thus, to provide a thorough study of the influence of various imaging conditions we compared not only imaging using different electron beam energies but also imaging of samples poststained using only osmium tetroxide, present in the freeze substitution solution, and also conventionally stained samples, using uranyl acetate and lead citrate.

Conventional high-voltage imaging was carried out on a transmission electron microscope Talos F200C (Thermo Fisher Scientific, Waltham, MA, USA), equipped with a Ceta-D Camera, using an electron beam voltage of 200 kV. Low-voltage images were obtained using a transmission electron microscope LVEM 25 (Delong Instruments, Brno, Czech Republic) equipped with an sCMOS camera BSI Teledyne for TEM mode, using a voltage of 25 kV.

Moreover, to explore the possibilities of reducing the chromatic aberration when imaging using low energies of the electron beam, the STEM mode of LVEM 25, using a YAG screen, followed by a photomultiplier for register STEM signal at 15 kV electron beam voltage, was employed as well as imaging at the same voltage using a scanning electron microscope Helios G4 HP (ThermoFisher Scientific, Waltham, MA, USA) equipped with a STEM3+ detector, while the bright field segment of the detector was selected for both microscopes.

## 3. Results

### 3.1. High Voltage Transmission Electron Microscopy

**Figure 2:**
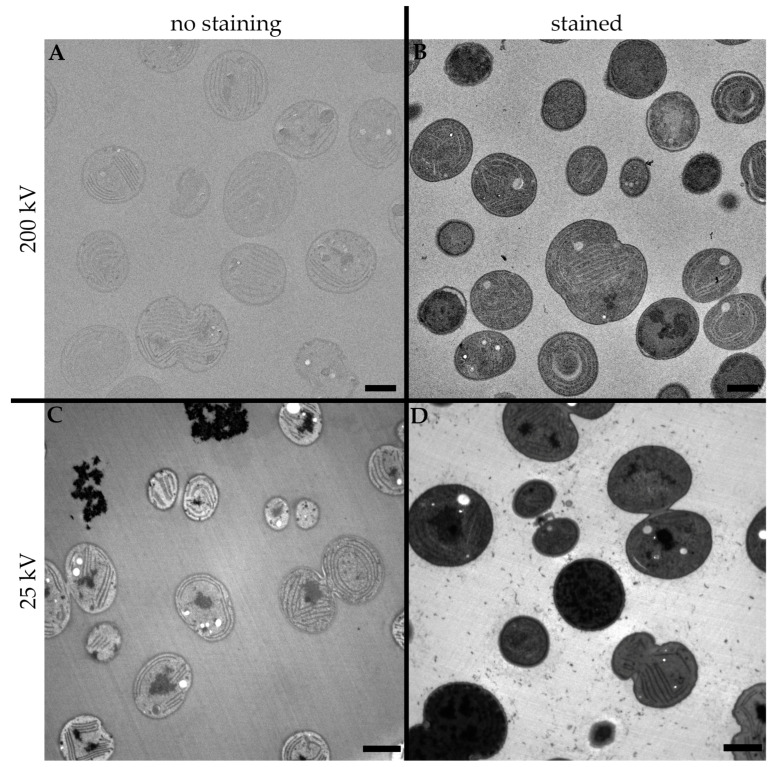
Comparison of the influence of different staining procedures and energies of the electron beam in the final TEM image on cyanobacterial cells. (**A**) A 200 kV accelerating voltage of electron beam without staining, (**B**) 200 kV accelerating voltage of electron beam and staining with uranyl acetate and lead citrate, (**C**) 25 kV accelerating voltage of electron beam without staining, (**D**) 25 kV accelerating voltage of electron beam and staining with uranyl acetate and lead citrate. Scale bar: 1 µm.

The influence of the staining procedure on conventional TEM imaging was substantial. As seen in Figure 2A, even though the OsO_4_ provided subtle contrast in the intracellular structures of the cyanobacterial cells, compared to the stained sample in Figure 2B, the ultrastructure is almost indistinguishable. 

**Figure 3:**
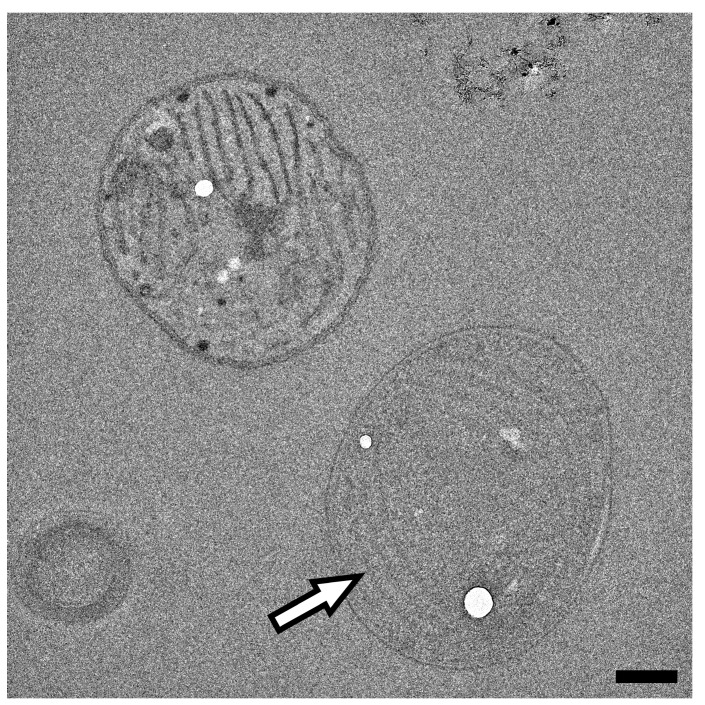
TEM image of unstained cyanobacterial cells obtained using a 200 kV electron beam. Thylakoid membranes marked by arrow. Scale bar: 500 nm.

Even the more detailed images of the unstained sample observed using a 200 kV electron beam provided minimal contrast with which to recognize the intracellular structures. As shown in Figure 3, the thylakoid membranes of the cyanobacterial cell are hardly distinguishable within the intracellular space and could even be considered ‘noise’ in the image, while other structures, possibly occurring in the cyanobacterial cells, are mostly unrecognizable.

**Figure 4:**
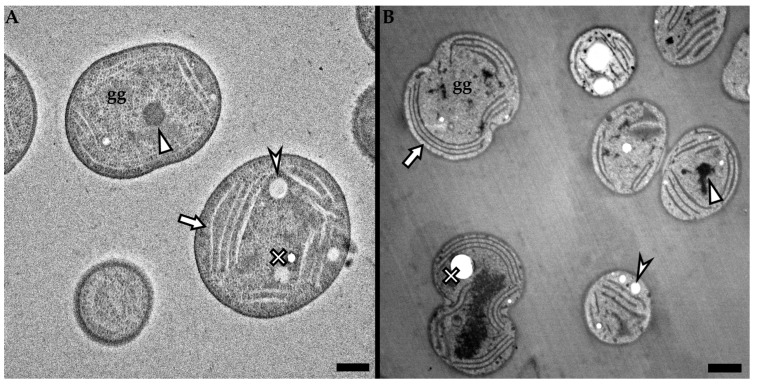
Intracellular structures of cyanobacterial cells were acquired using different imaging conditions. (**A**) Sample stained using uranyl acetate and lead citrate imaged using a 200 kV electron beam, (**B**) sample without staining imaged using a 25 kV electron beam. Intracellular structures marked by an arrow: thylakoid membranes; arrowhead: PHA granules; triangle: carboxysome; cross: washed-out granule; gg: glycogen granule. Scale bar: 500 nm.

On the other hand, when following the conventional procedure, the detailed images of the stained sample, observed using a 200 kV electron beam (Figure 4A), provide us with balanced contrast and, therefore, the intracellular structures such as thylakoid membranes (marked by arrow), carboxysome (marked by triangle), glycogen granules (marked by “gg”) are easily distinguishable, while it is even possible to recognize the difference between the electron-lucent PHA granules (marked by arrowhead) and the holes in the specimen owing to the remains of the washed-away granules (marked by cross) [46].

### 3.2. Low Voltage Transmission Electron Microscopy

As shown in the previous section, to obtain satisfactory contrasts in the images acquired by a high voltage transmission electron microscope, it is necessary to use contrasting agents, most commonly using uranyl acetate and lead citrate to stain ultrathin sections on the grid. However, if the accelerating voltage of the electron beam is lowered, the unstained sample (Figure 2C) provides an easily recognizable ultrastructure of the studied cells, which is, on the other hand, interestingly rather covered by the staining agents (Figure 2D), resulting in very dark images of the cells with hardly any distinguishable intracellular structures.

Furthermore, as seen in Figure 4B, the detailed image of an unstained sample observed using a 25 kV electron beam provides clearly recognizable intracellular structures, and in addition, when focusing on the resin surrounding the cells, the low voltage image provides minimum noise, whereby only the lines left by the knife during sectioning are slightly visible, while the glycogen granules are easily distinguishable from the ‘noise’.

### 3.3. Low Voltage Scanning Transmission Electron Microscopy

**Figure 5:**
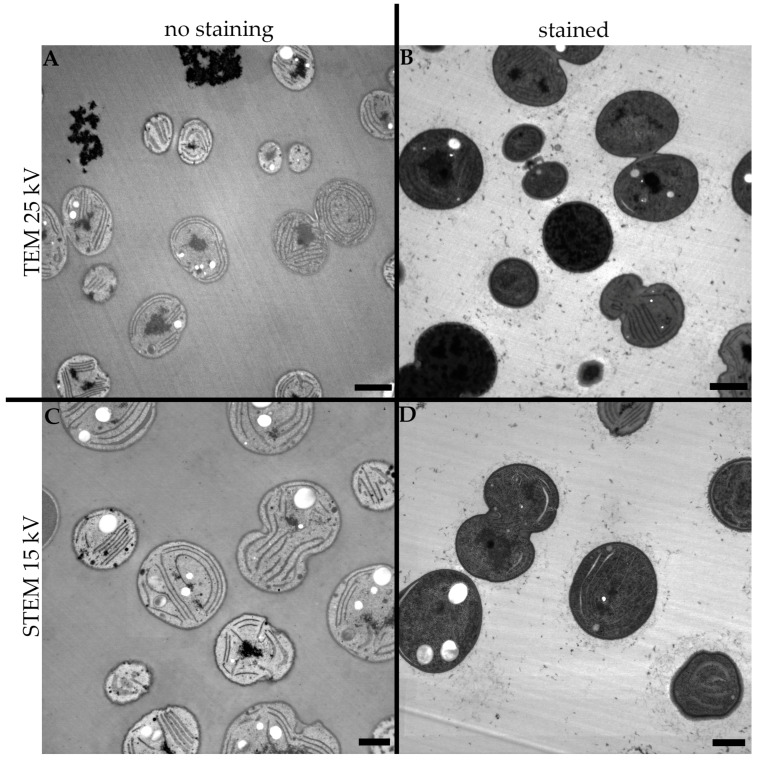
Comparison of the images of cyanobacterial cells obtained using a low voltage transmission electron microscope in different imaging modes. (**A**) Unstained sample imaged in TEM mode using a 25 kV accelerating voltage electron beam, (**B**) sample stained using uranyl acetate and lead citrate imaged in TEM mode using a 25 kV accelerating voltage electron beam, (**C**) unstained sample imaged in STEM mode using a 15 kV accelerating voltage electron beam, (**D**) sample stained using uranyl acetate and lead citrate imaged in TEM mode using a 15 kV accelerating voltage electron beam. Scale bar: 1 µm.

In scanning transmission electron microscopes (STEMs), the electron beam is focused on a very small probe and is scanned over the sample; for each position (pixel) transmitted electrons are recorded by a suitable STEM detector. Most TEM and scanning electron microscopes (SEMs), if they are equipped with the necessary hardware, can also enable STEM image recording. This technique is also very useful for imaging ultrathin sections of biological samples [47,48]. As shown in Figure 5C, the low-voltage STEM provides a sharp image of the ultrastructure of the cyanobacterial cells with a satisfactory contrast. Similarly to the low-voltage TEM, the stained sections of the samples provided a higher contrast than the unstained samples (Figure 5D). However, in the STEM images, it is still possible to recognize the elemental structures of the cells. 

**Figure 6:**
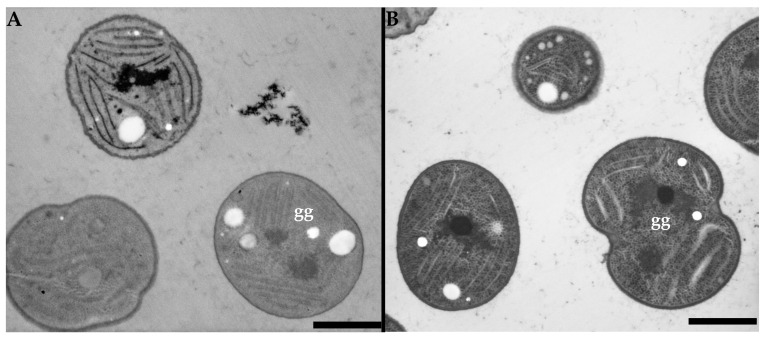
Cyanobacterial cells were imaged using SEM equipped with a STEM detector with a 15 kV accelerating voltage electron beam. (**A**) Unstained sample, (**B**) sample stained using uranyl acetate and lead citrate. Glycogen granules marked by “gg”. Scale bar: 1 µm.

To prove that the usefulness of the low-voltage imaging is not bound solely through the specialized low-voltage TEM, the same samples were also imaged using a SEM equipped with a STEM detector also operating at a 15 kV accelerating voltage. As seen in Figure 6A, the overall contrast of the unstained sample is comparable for both microscopes, while the specialized low-voltage TEM provided sharper images. However, the image of the stained sample (Figure 6B) shows more a balanced contrast than in the specialized low-voltage TEM, while the image is also sharper than in the unstained sample imaged by a SEM equipped with a STEM detector, which is demonstrated by the structure of glycogen granules.

**Figure 7:**
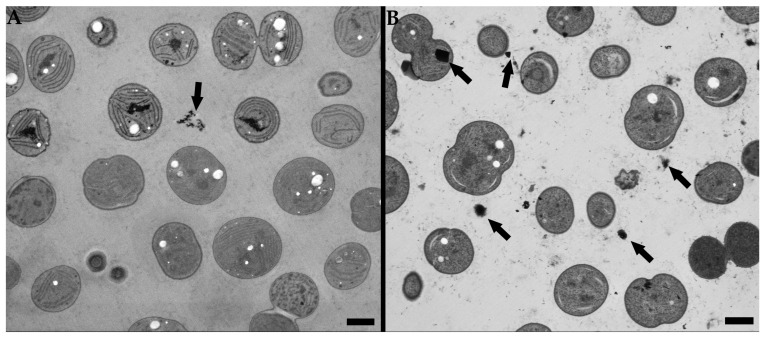
Cyanobacterial cells were imaged using SEM equipped with a STEM detector with a 15 kV accelerating voltage electron beam. (**A**) Unstained sample, (**B**) sample stained using uranyl acetate and lead citrate. Impurities marked by arrow. Scale bar: 1 µm.

Nevertheless, the problem of possible contamination of the sample by precipitated contrasting agents also remains for STEM imaging, similar to the risk for conventional TEM imaging. In Figure 7B, the impurities caused by the precipitated contrasting agents are clearly visible, as well as the impurities in Figure 7A, where the residual osmium tetroxide from the freeze substitution solution could have crystalized and left the electron-dense impurities in the sample. However, the extent of the contamination by the impurities is substantially higher for the conventional staining procedure than for the osmium-only staining.

## 4. Discussion

The comparison between the impact of the staining procedure and the accelerating voltage of the electron beam used for imaging the cyanobacterial cells shows substantial differences for each technique. The clear difference is already observed in the lower magnification images. As previously mentioned, the unstained samples observed using the 200 kV electron beam show almost no contrast; therefore, the ultrastructure of the cells is hardly visible. On the other hand, imaging the same unstained sample using low accelerating voltages of the electron beam provided a balanced contrast and, therefore, easily recognizable intracellular structures. 

If we would compare the conventional imaging method (specimen staining and imaging using a 200 kV electron beam) and the low voltage imaging without any on-section staining, the differences would be minimal. Both techniques provided us with detailed information on the intracellular structures of cyanobacterial cells. However, closer observations of the images show differences in the sharpness of the images and the presence of ‘noise’ in the detected signals. Images obtained using high voltages show the resin surrounding the cyanobacterial cells as grainy areas, suggesting the presence of noise from the detected signal in the image. As mentioned in Section 3.2., the unstained sample imaged using a low-voltage TEM shows minimum ‘noise’ in the detected signal, not only for the embedding resin but the glycogen granules inside the cells are also more clearly distinguishable compared to the conventionally stained and imaged sample. The ‘noise’ in the image of the conventionally stained and imaged sample is more apparent and, thus, the granules could be mistaken for the noise in the detected signal. Nevertheless, when observing the low-voltage TEM image, even though the overall contrast is well-balanced, some parts of the cells seem blurry and, therefore, the whole image is not as sharp as for the high-voltage TEM, a problem that can be overcome by several strategies, which will be discussed in following paragraphs. 

As mentioned above, with the benefits of low-voltage images also come the disadvantages, here, in the form of the need for very thin samples. Even though 20 nm-ultrathin sections were required for the 5 kV low-voltage TEM [25], this was overcome using a slightly higher voltage of 25 kV [26], although even the 25 kV imaging sometimes suffered from chromatic aberration. There were two sources of chromatic aberration when using electron microscopy. One of them occurs when the electron beam has a higher energy spread and results in the enlargement of the focal point and, therefore, in the loss of resolution in the final image. Using field-emission guns, which usually have narrow energy spreads, it is possible to diminish these defects in imaging. Another source of chromatic aberration emerges during the imaging of thicker samples using low-energy electrons, where inelastic scattering causes energy losses and, thus, an increase in the energy spread of the imaging electrons [49]. This phenomenon is visible in the low-voltage TEM images without staining. Even though the sections used for the imaging were ~70 nm thin, the cells in the image are a bit blurry. One option to overcome the effect of chromatic aberration is to cut even thinner sections than 70 nm, as was previously used with the 5 kV TEM [25]. However, there is also another option, which can eliminate the influence of chromatic aberration, obtain sharper images, and enable the circumvention of challenging sectioning. 

Scanning transmission electron microscopy is, as stated in Section 3.3, a very useful technique for biological samples. The advantage of STEM imaging lies with the focused beam scanned across the sample point by point, while transmitted electrons are detected for each point at different scattering angles. The image formation in STEM is less affected by the inelastic scattering occurring when imaging a thicker sample. Therefore, in STEM imaging the chromatic aberrations are less significant [50].

The difference is seen when imaging the same sample using low-voltage TEM and also STEM mode. Unstained cells imaged using STEM mode are sharper than those imaged in TEM mode while maintaining a balanced contrast and low ‘noise’ in the image. The same trend can also be seen for the sections stained with uranyl acetate and lead citrate. However, STEM imaging has some disadvantages in form of less comfortable imaging or higher beam damage to the sample by, for example, carbon contamination or mass loss [51,52]. 

That said, the problem with the resolution of the final images is not only affected by the voltage of the electron beam or the imaging mode. A crucial role is played by the microscope’s detector of the transmitted electrons. An example is shown in Section 3.3. The same samples observed by LVEM25 were also imaged using the SEM Helios G4 HP equipped with a STEM3+ detector of the transmitted electrons. Even though the contrast of the unstained specimen imaged using SEM equipped with a STEM detector was sufficient, the ultrastructure of the cyanobacterial cells was more easily distinguishable for the sample stained using uranyl acetate and lead citrate. The difference was seen the most in the structures of the glycogen granules. Compared with the images obtained using the STEM mode on the LVEM25 microscope, the details in the stained specimen are substantially more perceptible in an image acquired by the SEM equipped with a STEM detector than in the image acquired by a low-voltage TEM in the STEM mode. However, it is important to point out that the operator of the microscope can affect the settings for the contrast and brightness during the image acquisition and, therefore, also have an impact on the final outcome of the image. 

## 5. Conclusions

The study has proven that low-voltage (scanning) transmission electron microscopy offers great potential for the imaging of biological samples, in this case, cells of the cyanobacterium *Synechocystis* sp., PCC 6803. While high-voltage transmission electron microscopy requires staining of ultrathin sections using staining agents such as uranyl acetate and lead citrate, low-voltage transmission electron microscopy is capable of imaging the specimen without further staining. The problem with chromatic aberration occurring while imaging thicker samples can be overcome by cutting sections thinner than 70 nm, or by imaging the sample in the STEM mode of the microscope, if available. The overall results demonstrate that low-voltage electron microscopy could pose a method to replace conventional staining with highly toxic substances, such as uranyl acetate and it is worth further investigating the possibilities of LVEM.

## Data Availability

Not applicable.
